# What do psychiatrists think about primary mental health competencies among family doctors? A WPA–WONCA global survey

**DOI:** 10.1192/bji.2020.32

**Published:** 2021-02

**Authors:** R.M.K. Ng, T.F. Chan, H. Herrman, C. Dowrick

**Affiliations:** 1 Secretary for Education, World Psychiatric Association, Geneva, Switzerland. Email: rmkng@icloud.com; 2 Resident Trainee, Department of Psychiatry, Kowloon Hospital, Hong Kong, China; 3 President, World Psychiatric Association, Geneva, Switzerland; 4 Chair, Working Party on Mental Health, World Organization of Family Doctors (WONCA)

**Keywords:** Mental health competencies, World Psychiatric Association, World Organization of Family Doctors, primary care, psychiatric education

## Abstract

People with common mental disorders often seek medical attention from their family doctors. Thus, it is essential for family doctors to possess primary mental health knowledge. The aim of this study was to understand whether psychiatrists endorse the primary mental health competencies identified by the World Organization of Family Doctors and whether they agree that family doctors are demonstrating these competencies. A questionnaire was constructed based on 32 core competencies. Presidents of all World Psychiatric Association member societies were invited to complete the questionnaire or to forward it to local experts. According to the respondents, these competencies are considered relevant yet not sufficiently possessed by typical primary care doctors. Proposals are made to bridge this assumed competency gap.

One in four people suffers a mental health problem during their lifetime.^1^ The main burden of mental health problems is attributable to common mental disorders such as anxiety and depression.^2^ People suffering from anxiety or depression commonly present to their family doctors with mood problems, sleep disturbances or bodily discomfort.^3^ Therefore, family doctors are ideally positioned as the care providers for these patients. Furthermore, owing to stigma associated with psychiatry and limited access to psychiatrists,^4^ family doctors equipped with the knowledge and skills to assess and manage mental health problems are the most appropriate professionals to care for people with common mental disorders.^5^ A recent survey of educational needs of family doctors in the Asia Pacific Region carried out by the World Organization of Family Doctors (WONCA) has identified that 32% of respondents considered mental health as an educational priority.^6^ However, the exact core competencies that a family doctor needs to possess in order to deliver optimal care to these patients are yet to be agreed among different stakeholders.

If these core competencies of primary mental healthcare were clearly delineated, the training needs of family doctors for building up a strong primary mental health team could then be identified and addressed.^7^ The Working Party for Mental Health of WONCA recently released a list of core competencies based on a survey of a large group of family doctors using the Delphi method.^8^ Given that family doctors have a close working relationship with psychiatrists along the care pathways of patients with mental health problems, it would be interesting to understand whether or not psychiatrists endorse these core competencies. If psychiatrists regard these core competencies as important and relevant, it would be valuable from a training point of view to look into whether family doctors participating in mental healthcare are indeed demonstrating them.^9^ If certain core competencies are considered as highly important but distinctly absent among family doctors, this might invite further enquiry into the training needs in these competencies for family doctors.

As an international professional organisation representing more than 250 000 psychiatrists in over 140 countries around the world, the World Psychiatric Association (WPA) is dedicated to enhancing mental health education of all stakeholders involved in mental healthcare around the globe. The WPA therefore collaborated with WONCA in this endeavour of exploring psychiatrists’ opinions on the WONCA list of core competencies and whether primary care doctors possess them. The results of this survey will shed further light on future possible collaborative training initiatives with WONCA to enhance primary mental health competencies of family doctors in different parts of the world.

## Method

### Survey instrument

A survey questionnaire was constructed based on the list of core competencies in primary mental healthcare for family doctors. The list includes six domains of core competencies: (a) values – family doctors consider mental health as important; (b) communication skills – family doctors adopt person-centred approaches to assess, support and manage people with mental health problems; (c) assessment – family doctors diagnose and manage common mental health problems, can identify severe mental illness and assess risks; (d) management – family doctors manage common mental and physical health problems of people with severe mental illness; (e) collaboration and referral – family doctors mobilise a range of options and resources to support people with mental health problems and tailor them to patients’ and carers’ needs; and (f) reflective practice – family doctors take care of their own health and well-being. Under each domain, there are a number of expected skills and a knowledge base (for further details on the core competencies see WONCA Working Party on Mental Health^8^). These six domains contain 32 items, for each of which the respondent is invited to provide a dichotomous (‘yes/no’) answer to two questions: (a) ‘Do you agree that these competencies can reasonably be expected of trained and qualified family doctors?’; (b) ‘To what extent do family doctors in your region currently demonstrate these competencies?’. There is an additional open-ended question about what the respondent thinks could be done to help family doctors expand their competencies if needed. The questionnaire took around 15–20 min to complete. Both English and Spanish versions were available.

### Sample recruitment

The presidents of all WPA member societies were invited online to complete this questionnaire between September 2018 and April 2019. The presidents were requested to forward the questionnaire to local experts with primary mental health experience if they considered themselves as not the appropriate ones to respond to it. Each society was requested to respond only once. It is an established practice that the results of all WPA surveys approved by the WPA executive committees are shared among all relevant WPA stakeholders in the form of newsletters, position statements, recommendations and reports available on the WPA official website.

### Data analyses

All quantitative data were analysed using SPSS Version 23 for Windows. The frequency and percentages of responses to the questions requiring dichotomous (‘yes/no’) answers were tabulated. For qualitative data obtained from the open-ended questions, the answers were entered word-by-word in English into the analytical NiVo software (for Windows) for qualitative analyses. Themes and subthemes generated were then examined and reviewed by R.M.K.N. for their relevance and appropriateness to the open-ended questions.

## Results

**Fig. 1:**
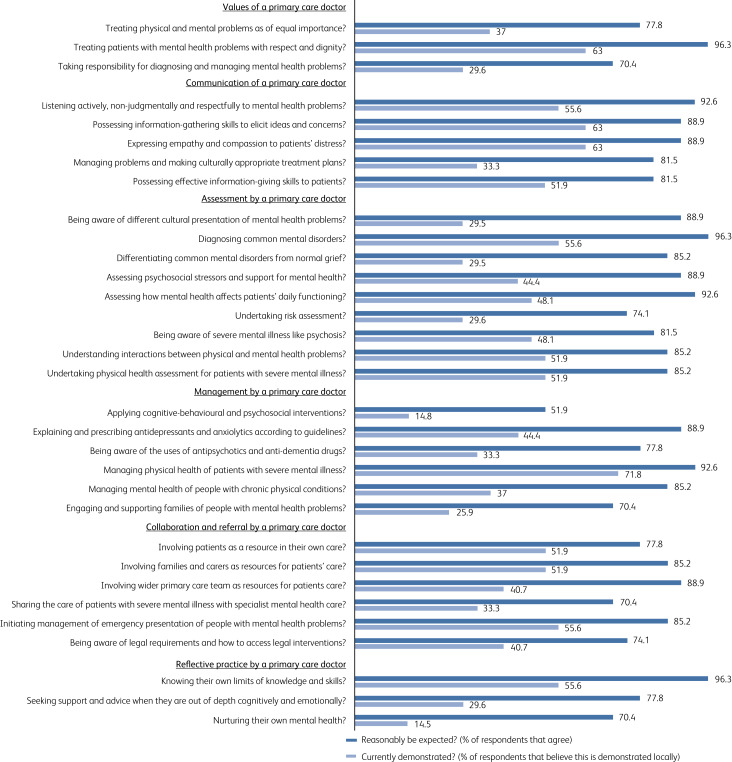
Responses of WPA member societies (*n* = 31) to the 32 survey questions on core competencies in mental health for primary care doctors.

Thirty-one member societies (22% of 142 WPA member societies) from 28 countries responded to the survey. The responding societies represent 65% of psychiatrists around the globe, as assessed by membership numbers declared to the WPA. The proportion of participating WPA member societies from different regions of the world is as follows: 41% (*n* = 12) from Asia, including Australia and New Zealand; 31% (*n* = 10) from North and South America; and 29% (*n* = 9) from Europe. There was no response from member societies from Africa. Forty-one percent (*n* = 13) of the respondents identified themselves as presidents of the WPA member societies and the remainder as local experts in the field of primary mental healthcare. Forty-one percent (*n* = 13) of the responding societies represent low- and middle-income countries as defined by the World Bank. A break-down of responses to the survey questions is shown in Fig. 1. All core competencies except one (applying cognitive–behavioural therapy and psychosocial interventions) were considered by 70% (*n* = 22) of respondents to be relevant and important for primary care doctors. Eight of these core competencies were rated by less than 30% of respondents as being demonstrated by primary care doctors in their locality.

Thematic analyses of the narratives given by the respondents on ways of improving the acquisition of these core competencies in mental health for primary care doctors came up with the following themes: (a) training and education at undergraduate, postgraduate and specialist levels with emphasis on developing proper attitudes, skills and knowledge; (b) fundamental changes in clinical team structure so that psychiatrists can work closely with primary care doctors in various models, including primary mental health teams, visiting psychiatrists in primary care clinics, use of guidelines in primary care clinics and setting up of group reflective practice; (c) fundamental changes at an organisational level to allow minimum protected time for each primary care consultation, providing incentive for primary care doctors to treat mental health problems, providing an environment that promotes work–life balance. In addition, WONCA members may work with respective national psychiatric societies to organise mental well-being training and facilitate a confidential referral system of mental health assessment and intervention for primary care doctors suffering from mental health distress; (d) systematic change at a national level so that primary care doctors are legally allowed to diagnose and treat common mental disorders, national mental health insurance available for all walks of life, a national anti-stigma campaign about mental health problems rolled out across the whole country, and public education to empower patients and carers to demand primary mental healthcare and parity of care for physical and mental health problems. The overarching theme that emerged is that developing these core competencies requires changes at individual, clinical team, hospital and national levels of healthcare. These changes must be implemented in a concerted way to bring about the suggested improvements.

## Discussion

This is the first collaborative study conducted jointly by the World Psychiatric Association (WPA) and the World Organization of Family Doctors (WONCA). With increasing evidence that a substantial proportion of the global disease burden is attributable to mental health problems, doctors working in primary healthcare will be expected increasingly to play an important role in providing appropriate mental healthcare to patients who present commonly with somatic health problems and live in the communities served by these doctors. The core competencies proposed by the Working Party for Mental Health of WONCA were considered relevant, appropriate and important by most of the experienced and senior psychiatrists from different parts of the world who participated in this study.

The key challenge is that, in the eyes of these psychiatrists, the typical primary care doctor does not possess these competencies. The perceived lack of these core competencies may be due to lack of communication between psychiatrists and primary care doctors in the respective localities. It may be that psychiatrists simply are unaware of the knowledge and skills already possessed by their primary care counterparts. This hypothesis is proposed because many respondent psychiatrists considered reflective practice as highly important, yet believed that primary care doctors do not demonstrate it. Reflective practice as a core competency for a doctor in any specialty or setting involves being mindful and aware of moment-to-moment changes in their own practice, as well as reflecting on what they have done well and what they could have done better. Such reflective practice usually leads to steps seeking improvement in their clinical practice.^10^ It is not clear how and why respondent psychiatrists would have the perception that most primary care doctors do not possess skills in reflective practice. This warrants further enquiry.

Assuming that a number of these important and relevant core competencies are indeed not possessed by many primary care doctors, the suggested improvement measures proposed by the respondent psychiatrists seem to be highly reasonable and feasible. In what ways can the WPA work with WONCA in implementing some of these improvement measures? The two organisations are now planning to work with their respective national member societies to form a unified voice to lobby local governments to implement changes at a national level. Pilot work will be conducted in one or two representative countries on each continent to form a joint WPA–WONCA platform to lobby the local medical regulatory authorities and university deans to incorporate mental health education into medical school curricula. Besides, the two organisations are working together on The Lancet–WPA Commission on Depression,^11^ which gives the opportunity to develop and promulgate the most up-to-date evidence-based materials on depression to members of the WPA and WONCA through their online educational learning platforms. In future, the two organisations will also engage other stakeholders, such as universities, training colleges and policy makers, to focus on research and policy issues.

### Limitations

The major limitation of this study is the low response rate from WPA member societies. The responding societies, however, represent more than half of the psychiatrists worldwide recorded by the WPA. The second limitation is that the views of the national society presidents and local experts do not necessarily represent the majority views of the psychiatrists working in their respective countries. Nevertheless, given their knowledge and experience of the service models and workforces in their own mental health systems, their views at least represent the perception of influential groups of senior local psychiatrists on the roles and competencies of primary care doctors in mental health. The third limitation is the lack of response from member societies from Africa. R.M.K.N. made informal contact with presidents of several member societies in Africa and they gave three main reasons for their lack of response. In their view: (a) primary care doctors do not exist in significant numbers in their countries; (b) few patients with mental health problems would choose to see a primary care doctor; and (c) primary care doctors are busy with patients with physical health problems and do not have time and interest to take care of patients with mental health problems. The final limitation is that this study tapped into the views of senior psychiatrists in the respective countries and may not reflect the views or experiences of family doctors themselves. Understanding these is a separate and important enquiry of concern to professionals, communities and healthcare systems around the world.

### The future

Influential groups of psychiatrists in WPA member societies across three of the four world regions have a deep interest in and commitment to working with family care doctors to increase access to high-quality mental healthcare in the communities. The WPA and WONCA have started working together to support their member societies to collaborate in their countries. They can then find and evaluate locally relevant ways, through training, practice and other means, to support the development of core competencies in mental health in primary healthcare.

## Supplementary material

For supplementary material accompanying this paper visit https://doi.org/10.1192/bji.2020.32. click here to view supplementary material
